# Discrete Choice Experiment to Evaluate Factors That Influence Preferences for Antibiotic Prophylaxis in Pediatric Oncology

**DOI:** 10.1371/journal.pone.0047470

**Published:** 2012-10-17

**Authors:** Dean A. Regier, Caroline Diorio, Marie-Chantal Ethier, Amanda Alli, Sarah Alexander, Katherine M. Boydell, Adam Gassas, Jonathan Taylor, Charis Kellow, Denise Mills, Lillian Sung

**Affiliations:** 1 Canadian Centre for Applied Research in Cancer Control, British Columbia Cancer Research Agency, Vancouver, British Columbia, Canada; 2 Program in Child Health Evaluative Sciences, The Hospital for Sick Children, Toronto, Ontario, Canada; 3 Division of Haematology/Oncology, The Hospital for Sick Children, Toronto, Ontario, Canada; 4 Community Health Systems Resource Group, The Hospital for Sick Children, Toronto, Ontario, Canada; 5 Program in Health Research Methodology, McMaster University, Hamilton, Ontario, Canada; University of Massachusetts Medical School, United States of America

## Abstract

**Background:**

Bacterial and fungal infections in pediatric oncology patients cause morbidity and mortality. The clinical utility of antimicrobial prophylaxis in children is uncertain and the personal utility of these agents is disputed. Objectives were to use a discrete choice experiment to: (1) describe the importance of attributes to parents and healthcare providers when deciding between use and non-use of antibacterial and antifungal prophylaxis; and (2) estimate willingness-to-pay for prophylactic strategies.

**Methods:**

Attributes were chances of infection, death and side effects, route of administration and cost of pharmacotherapy. Respondents were randomized to a discrete choice experiment outlining hypothetical treatment options to prevent antibacterial or antifungal infections. Each respondent was presented 16 choice tasks and was asked to choose between two unlabeled treatment options and an opt-out alternative (no prophylaxis).

**Results:**

102 parents and 60 healthcare providers participated. For the antibacterial discrete choice experiment, frequency of administration was significantly associated with utility for parents but not for healthcare providers. Increasing chances of infection, death, side effects and cost were all significantly associated with decreased utility for parents and healthcare providers in both the antibacterial and antifungal discrete choice experiment. Parental willingness-to-pay was higher than healthcare providers for both strategies.

**Conclusion:**

Chances of infection, death, side effects and costs were all significantly associated with utility. Parents have higher willingness-to-pay for these strategies compared with healthcare providers. This knowledge can help to develop prophylaxis programs.

## Introduction

Bacterial and fungal infections continue to be common causes of mortality for children receiving intensive chemotherapy. [1] Infections also cause morbidity, limit the ability to deliver anti-cancer therapy, decrease quality of life, and are associated with substantial costs. [2] A meta-analysis of randomized trials of prophylactic antibacterial medication in neutropenic adult oncology patients showed a significantly decreased risk of death in patients receiving prophylaxis. [3] Similarly, a meta-analysis of randomized controlled trials demonstrated that antifungal prophylaxis significantly decreased all-cause mortality in patients (mainly adults) receiving chemotherapy. [4] The evidence is much more limited in children with cancer and we have previously shown substantial variation in practices related to antibacterial and antifungal prophylaxis within North American and Germany for children with acute myeloid leukemia [5].

Preferences toward antibiotic prophylaxis may, in part, explain such variability in practice. One method for measuring preferences in the face of multiple trade-offs in health care is the discrete choice experiment (DCE) methodology. [6] The conceptual basis of DCE begins with the assumption that health care “goods” can be described by their characteristics (called attributes) and that individuals derive value (utility) from these attributes. [7] In the present example, the decision to accept routine antibiotic prophylaxis may be influenced by several factors such as the risks of invasive infection, infectious death and drug toxicity, and the requirement to give the medication intravenously or orally. In addition, costs may play a role in decision making. Using a DCE, it is possible to quantify the importance and relative importance of each factor that is hypothesized to drive the decision to receive routine prophylaxis. Including a cost attribute in the DCE allows for a monetary measure of utility called willingness-to-pay (WTP). The objectives were to use a DCE to describe the importance of attributes to parents and healthcare providers (HCPs) when deciding between use and non-use of antibacterial and antifungal prophylaxis, and to estimate WTP for prophylactic strategies.

**Table 1:** Attributes and attribute levels used in the discrete choice experiment.

	Bacterial Infection:Prophylaxis	Bacterial Infection:No Prophylaxis	Fungal Infection:Prophylaxis	Fungal Infection:No Prophylaxis
Risk of Infection (%)	10, 30, 50, 70	10, 30, 50, 70	1, 3, 5, 10	1, 3, 5, 10
Risk of Death (%)	0.1, 1, 3, 5	0.1, 1, 3, 5	0.1, 1, 3, 5	0.1, 1, 3, 5
Risk of nausea, vomiting, diarrheaor headache (%)	5, 15, 25, 50	5, 15, 25, 50	5, 15, 25, 30	5, 15, 25, 30
Route of administration	1 oral OD	No medication	1 oral OD	No medication
	1 oral BID		1 oral BID	
	2 oral: 1 TID		1 IV OD	
	and 1 BID		1 IV BID	
Cost	$100.00	$0	$300.00	$0
	$200.00		$3000.00	
	$400.00		$6000.00	
	$700.00		$7000.00	

Abbreviations: OD – once daily; BID – twice daily; TID – three times daily; IV – intravenous.

## Methods

### Ethics Statement

This study was approved by The Hospital for Sick Children Research Ethics Board. All participants consented to participation in writing and demographic information was obtained at this time. Additional information about the children of parent participants, including diagnosis and treatment information, were abstracted from the child’s chart.

**Figure 1:**
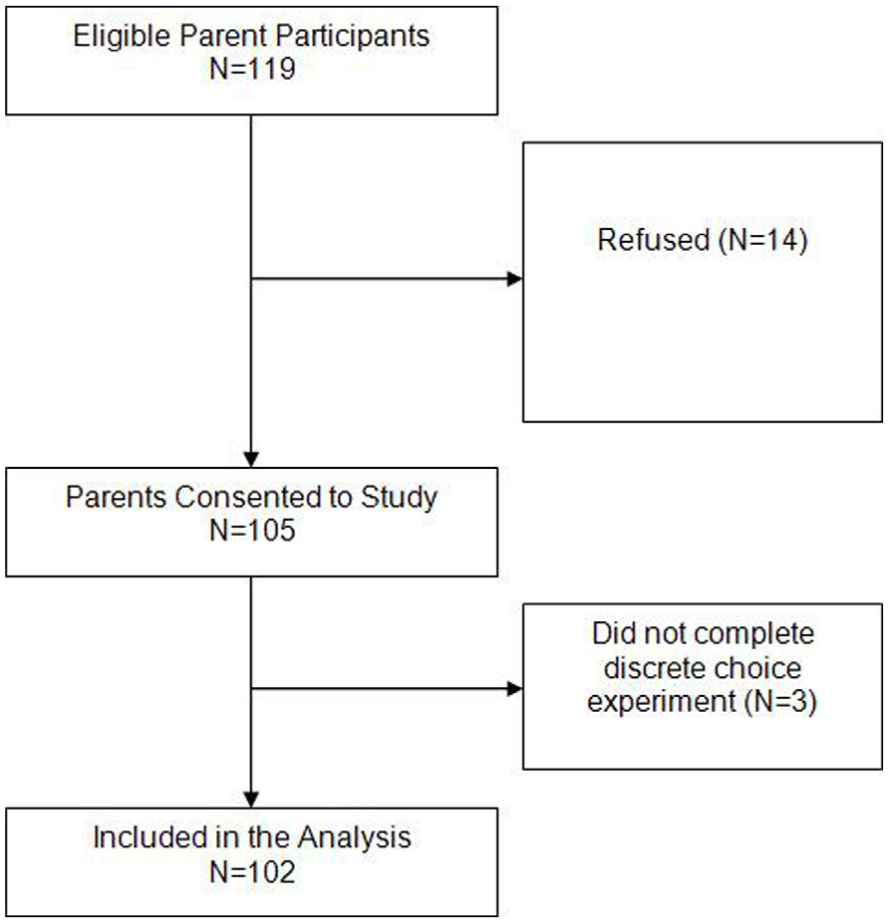
Flow diagram of parent participants through the study.

**Table 2:** Demographics of the parents in the study cohort.

	Total Cohort N = 102	Bacterial DCE N = 52	Fungal DCE N = 50
**Parent Characteristics**			
Median age in years (IQR)	40.0 (36.6, 44.3)	40.0 (36.5, 42.0)	42.0 (36.0, 45.0)
Male (%)	26/101 (25.5)	12 (23.1)	14/49 (28.6)
Married (%)	89/101 (88.1)	44 (86.3)	44/49 (89.8)
Education (%)			
Professional/graduate	17 (16.7)	11 (21.2)	6 (12.2)
College/university	67 (65.7)	32 (61.5)	35 (71.4)
High school	15 (14.7)	7 (13.5)	8 (16.3)
Primary/middle school	0 (0.0)	0 (0.0)	0 (0.0)
Other	1 (1.0)	1 (2.0)	0 (0.0)
Not reported	2 (2.0)	1 (2.0)	1 (2.0)
Full time employment (%)	56/100 (56.0)	27/51 (52.9)	29/49 (59.2)
Other health plan (Besides OHIP) (%)	81/99 (81.8)	40/51 (78.4)	41/48 (85.4)
Annual income ≥ $60,000 (%)	50 (49.0)	26 (50.0)	24 (48.0)
**Characteristics of their Children**			
History of FN (%)	53/101 (52.5)	28/51 (54.9)	25 (50.0)
Cancer type (%)			
Brain tumor	8 (7.8)	2 (3.8)	6 (12.0)
Leukemia/other hematological malignancies	47 (46.1)	25 (48.1)	22 (44.0)
Lymphoma	15 (14.7)	7(13.5)	8 (16.0)
Solid tumor	32 (31.4)	18 (34.6)	14 (28.0)
Relapsed cancer (%)	11 (10.8)	7 (13.5)	4 (8.0)

Abbreviations: IQR – interquartile range; FN – fever and neutropenia; OHIP – Ontario Health Insurance Plan; DCE – discrete choice experiment.

### Participants

There were two groups of respondents: (1) HCPs working with children with cancer including physicians, nurse practitioners, pharmacists and social workers at a large tertiary care pediatric cancer center, The Hospital for Sick Children (SickKids), Toronto, Canada; and (2) Parents of children ages 0 to 18 years receiving active treatment for cancer at SickKids. We excluded respondents unable to read English.

**Table 3:** Multinomial conditional logit regression illustrating influence of attributes on utility in the antibacterial prophylaxis sencario.

	Entire Cohort n = 82 (95% CI)	Parents n = 52 (95% CI)	HCP n = 31 (95% CI)
Route of Administration			
One oral OD	0.17* (−0.03,0.30)	0.24* (0.07,0.42)	−0.09 (−0.35,0.16)
One oral BID	0.06 (−0.04,0.30)	0.06 (−0.11,0.22)	0.19 (−0.02,0.41)
Two oral: one TID and one BID	−0.23* (−0.37, −0.10)	−0.30* (−0.49,−0.12)	−0.10 (−0.32,0.12)
Chance of Infection	−4.17* (−4.95,−3.40)	−4.34* (−5.04,−3.64)	−4.43* (−5.33,−3.54)
Chance of Death	−47.24* (−58.38,−36.11)	−45.76* (−53.75,−37.76)	−62.10* (−74.96, −50.22)
Chance of a Side Effects	−1.99* (−2.87,−1.10)	−1.50* (−2.30,−0.71)	−3.21* (−4.31,−2.11)
Cost per 28 Days of Treatment(hundreds $CDN)	−0.10* (−0.15,−0.06)	−0.08* (−0.14,−0.02)	−0.15* (−0.22,−0.07)
Alternative Specific Constant for Prophylaxis	0.22 (−0.06, −0.50)	0.29 (−0.10,0.68)	0.44 (−0.04,0.93)
Pseudo R2	0.34	0.37	0.36

* Significant at 5% level. Table represents β coefficients (95% confidence interval) and P values from multinomial conditional logit regression. The regression coefficients for each attribute level represent the mean part-worth utility of that attribute level in the respondent sample. Pseudo R2 reflects model fit with higher pseudo R2 reflecting better fit.

Abbreviation: HCP – healthcare provider; CI – confidence interval; BID – twice daily; TID – three times daily.

**Table 4:** Multinomial conditional logit regression illustrating influence of attributes on utility in the antifungal prophylaxis scenario.

	Entire Cohort; n = 79 (95% CI)	Parents; n = 50 (95% CI)	HCP; n = 29 (95% CI)
Route of Administration			
One oral OD	0.26* (0.11,0.42)	0.21* (0.02,0.39)	0.40* (0.12,0.68)
One oral BID	−0.04 (−0.20,0.12)	−0.01 (−0.21,0.18)	−0.13 (−0.43,0.16)
One IV OD	0.02 (−0.13,0.18)	−0.04 (−0.23,0.15)	0.20 (−0.07,0.48)
One IV BID	−0.25 (−0.39, −0.08)	−0.15 (−0.33,0.04)	−0.47* (−0.76, −0.16)
Chance of Infection	−13.03* (−16.01,−10.05)	−12.72* (−16.32,−1.73)	−14.44* (−19.87,−9.01)
Chance of Death	−46.66* (−52.42,−40.91)	−41.31* (−48.14,−34.60)	−60.07* (−71.01,−46.40)
Chance of a Side Effects	−2.65* (−3.65,−1.66)	−2.96* (−4.18,−1.73)	−2.00* (−3.74,−0.26)
Cost per 28 Days of Treatment(hundreds $CDN)	−0.02* (−0.2,−0.02)	−0.014* (−.02, −.01)	−0.03* (−.04, −.0.02)
Alternative Specific Constant for Prophylaxis	−0.58* (−0.90,−0.26)	−0.41* (0.80,0.02)	−0.98* (−1.56, −0.40)
Pseudo R2	0.24	0.22	0.30

* Significant at 5% level. Table represents β coefficients (standard error) and P values from multinomial conditional logit regression. The regression coefficients for each attribute level represent the mean part-worth utility of that attribute level in the respondent sample. Pseudo R2 reflects model fit with higher pseudo R2 reflecting better fit.

Abbreviation: HCP – healthcare provider; CI – confidence interval; OD – once daily; BID – twice daily; IV - intravenous.

During the time frame of the study, children at SickKids did not receive routine antibacterial prophylaxis (other than co-trimoxazole, which was used for *Pneumocysitis jirovecii* prophylaxis). Fluconazole (antifungal prophylaxis) was administered for patients with acute myeloid leukemia, relapsed acute lymphoblastic leukemia, and those undergoing hematopoietic stem cell transplantation.

**Table 5:** Willingness-to-pay for antibacterial and antifungal prophylaxis*.

	Entire Cohort	Parents (95% CI)	HCP (95% CI)
**Antibacterial Prophylaxis**			
Prophylaxis scenario	$994 (753,1511)	$1,504 (959,3729)	$717 (504,1259)
1% reduction chance of infection	$40 (28,64)	$53 (31,146)	$29 (18,56)
1% reduction chance of death	$448 (312,739)	$558 (316,1498)	$404 (260–774)
1% increase in chance of side effects	$−19 (−32, −12)	$−18 (−51,−8)	$−21 (−51,−12)
**Antifungal Prophylaxis**			
Prophylaxis scenario	$1,417 (840,2008)	$2,146 (466,4773)	$735 (75,1397)
1% reduction chance of infection	$241 (177,320)	$316 (210,470)	$169 (104,249)
1% reduction chance of death	$968 (778,1227)	$1,135 (818,1669)	$821 (624,1088)
1% increase in chance of side effects	$−47 (−68, −28)	$−70 (−114,−38)	$−22 (−42,−3)

* Willingness-to-pay for each scenario (95% confidence interval). Antibacterial prophylaxis scenario consists of the following for chances of infection, death and side effects for prophylaxis versus no prophylaxis: 25%, 1% and 25% versus 50%, 2%, 15%. Antifungal prophylaxis scenario consist of the following chances of infection, death and side effects for prophylaxis versus no prophylaxis: 3%, 2% and 25% versus 10%, 5% and 15%.

Abbreviation: HCP – healthcare provider; CI – confidence interval.

### Identification of Attributes, Questionnaire Design, and Administration

The attributes and levels used to describe prophylactic treatments were informed through literature review and subsequent qualitative interviews with three experienced pediatric oncology physicians at SickKids. The levels of each attribute were selected to accommodate a range of actual and theoretical clinical outcomes associated with bacterial or fungal infections under prophylactic and no prophylactic strategies. The attributes identified were chances of infection, death and side effects, route of administration and cost of pharmacotherapy (see accompanying article). We displayed common side effects including nausea, vomiting, diarrhea and headache. Costs were for 28 days of treatment and were stated to be out-of-pocket and not covered by insurance, since the Canadian healthcare system does not routinely cover outpatient medication costs. We did not include antimicrobial resistance as an attribute because the qualitative interviews indicated that resistance was thought to be important at a guideline/general recommendation level but not important for individual patient decision making. A pilot study then was conducted with parents and HCPs who met eligibility criteria (n = 9) to ensure that the attributes and levels informing each choice task were relevant, clear and comprehensible. The final attributes and their levels are illustrated in Table 1.

**Figure 2:**
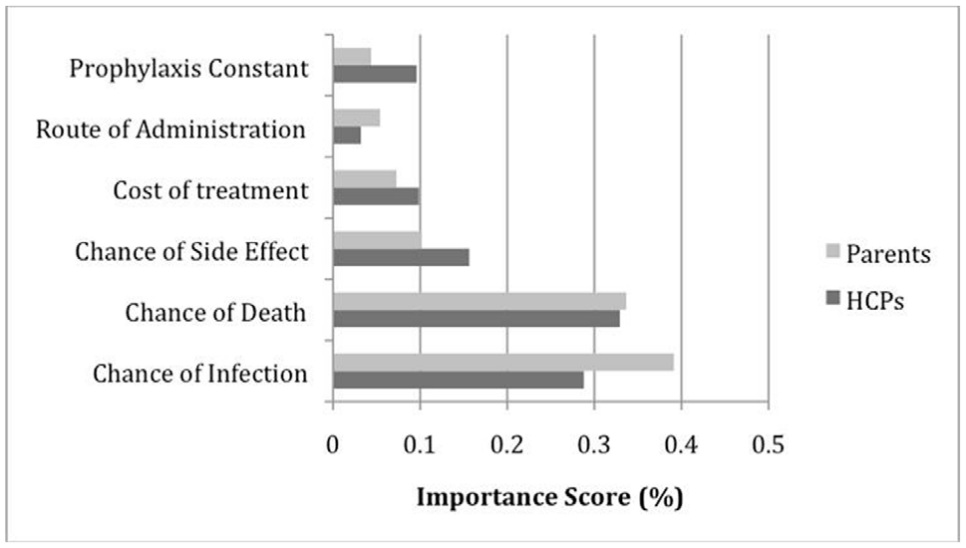
Attribute importance ranking for antibacterial prophylaxis. Abbreviation: HCPs – healthcare providers.

**Figure 3:**
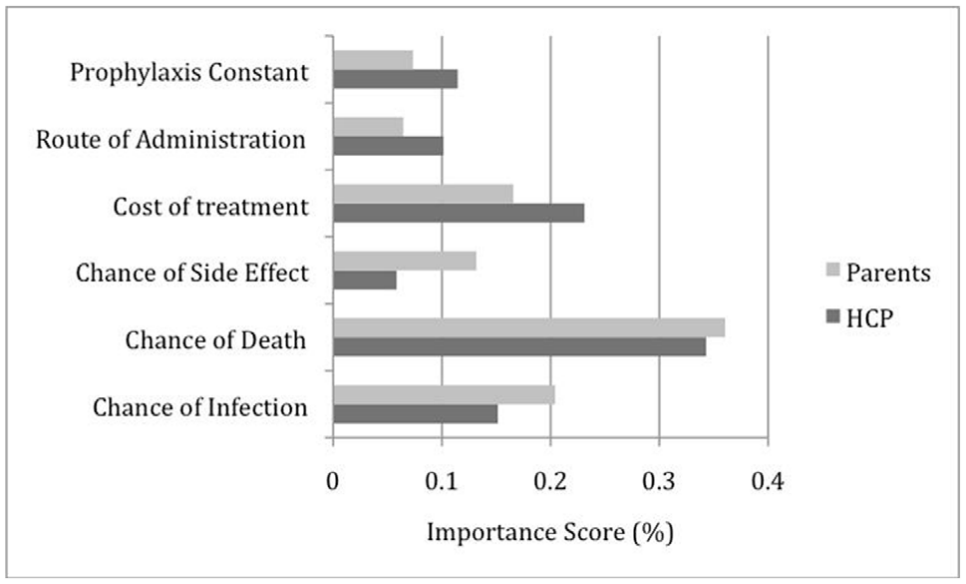
Attribute importance ranking for antifungal prophylaxis.

The pilot study indicated that completion of both the bacterial and fungal DCE would be excessively burdensome for respondents. Respondents were therefore randomized to a DCE outlining hypothetical treatment options to prevent either bacterial or fungal infections. Each respondent was presented with a standardized outline of bacterial and fungal infection including a description of risks, symptoms and possible health outcomes. Antibacterial or antifungal prophylaxis was then described without reference to a specific agent. Respondents were asked to imagine that their child/patient was a candidate for prophylaxis. Two unlabeled treatment alternatives and a no prophylaxis treatment option were then described using visual aids. The choice tasks varied the attribute levels of the options to gain an understanding of how respondents’ preferences change depending on the attribute levels. The choice tasks were generated using a D-optimal approach designed to elicit the maximum information from respondents. [8] Each respondent was sequentially presented with 16 choice tasks on flash cards with information in both numeric and visual formats (Appendix S1), and was asked to choose between the two treatment options and the opt-out alternative. The choice tasks were presented by trained interviewers using standardized scripts and props.

### Statistical Models

Multinomial conditional logit regression was employed to analyze the effect of the attribute levels and no medication on parents’ and HCPs’ preferences (split sample analysis). Each DCE attribute was included in the regression model. The ‘no prophylaxis medication’ alternative was included as an alternative specific constant to account for any latent or uncontrolled factors when choosing the no prophylaxis alternative. Effects coding was used to represent the route of administration attribute, and the risks of death, infection and side effects were coded as continuous variables. The reference levels for route of administration were represented by 2 oral medications (1 three times a day and 1 twice daily) for the antibacterial prophylaxis DCE and intravenous twice daily for the antifungal prophylaxis DCE. The no prophylaxis medication alternative was coded such that when respondents had an overall preference to have prophylaxis medication (after controlling for the included attribute levels), this parameter would be positive. The cost attribute was scaled in hundreds of Canadian dollars. The regression coefficients for each attribute level represent the mean part-worth utility of that attribute level in the respondent sample. Differences in the scale parameter prevent direct comparison of utility part-worth between respondent subgroups; [9] indirect comparisons were examined through attribute importance, which calculates the relative importance of each attribute to each respondent subgroup such that the importance values of the attributes add to 100% [10].

In economics, WTP is a monetary value of the change in utility or welfare of a respondent if they were to secure the expected benefit the technology offers. Average WTP can be interpreted as an overall measure of the change in welfare (benefit) to respondents. This study examined the average marginal WTP for each attribute in each DCE followed by average WTP for prophylaxis versus no prophylaxis. The WTP for prophylaxis should take into account the probabilistic nature of the regression model and the likelihood that respondents found the scenario representing prophylaxis acceptable. The multiple alternatives formula was employed, [11] and the average WTP estimate and its 95% confidence interval (CI) were derived using Monte Carlo simulation techniques.

Calculating average WTP for prophylaxis treatment requires the definition of a scenario representing the value of the attributes for treatment with antibacterial or antifungal prophylaxis. For antibacterial prophylaxis, the chances of infection, death and side effects were assumed to be 25%, 1% and 25% respectively, and 50%, 2%, and 15% for no prophylaxis. For antifungal prophylaxis, the chances of infection, death and side effects were 3%, 2%, and 25% for prophylaxis and 10%, 5%, and 15% for no prophylaxis.

## Results

Between June and October 2011, 119 eligible parent participants were identified; 14 refused and 3 did not complete the DCE, leaving a total of 102 parents (Figure 1). Sixty-one HCPs were identified; one did not complete the DCE, leaving 60 HCPs. Table 2 illustrates the demographics of the parent cohort and those randomized to the bacterial and fungal DCE. For the HCP cohort, 31 were randomized to the bacterial DCE and 29 to the fungal DCE. Together, 17 (28.3%) HCPs were male with a median age of 40.0 (interquartile range (IQR) 32.0, 43.0) years and a median of 8.0 (IQR 2.9, 15.0) years of experience. Roles included staff physician (12, 20.0%), fellow (26, 43.3%), nurse practitioner (9, 15.0%), pharmacist (9, 15.0%) and social worker (4, 6.7%).

Tables 3 and 4 illustrate the marginal effect of each attribute on utility (utility part-worth) for antibacterial or antifungal prophylaxis. In other words, the regression coefficients in these tables indicate the degree to which attribute levels affect stated well-being in the context of prophylaxis. In the antibacterial prophylaxis DCE, increasing chances of infection, death, and side effects, and cost of treatment were statistically significantly associated with decreased utility. Overall, parents’ and HCPs’ alternative specific constants were positive, which indicates they preferred antibacterial prophylaxis after controlling for each of the attribute levels. For parents, taking an oral medication once daily was significantly associated with higher utility, and taking two medications with five administrations per day was significantly associated with worse utility. The frequency of oral administration did not significantly influence utility among HCPs.

The antifungal prophylaxis analysis (Table 5) demonstrated that for parents and HCPs, increasing chances of infection, death, side effects and cost per 28 days of treatment were significantly associated with decreased utility. Parents and HCPs had a statistically significant preference for no antifungal prophylaxis after controlling for all the attribute levels. For both parents and HCPs, oral administration once daily was associated with better utility.

Figures 2 and 3 summarize the attribute importance between respondent groups for each DCE. HCPs exhibited stronger negative preference compared to parents for spending money on antibacterial or antifungal prophylaxis. Parents exhibited stronger positive preference for decreased chances of infection and death in both DCEs. For parents and HCPs, the three most important attributes for antibacterial prophylaxis were chances of infection, death, and side effects. The importance ranking, however, differed between the attributes with parents placing more importance on chance of infection and HCPs giving greater importance to chance of death. In the antifungal DCE, the importance rank order for the top three attributes were chances of death and infection, and cost of treatment for both respondent groups.

Table 5 illustrates the WTP estimates for prophylaxis. For the antibacterial prophylaxis scenario, parents and HCPs were willing to pay $1504 (95% CI $959, $3729) and $717 (95% CI $504, $1259), respectively; there was no statistically significant difference in WTP between the two group (P = 0.56). The marginal willingness estimates (accounting for scenario ‘uptake’) for parents and HCPs were $53 and $29 for a 1% reduction in chance of infection, $558 and $404 for a 1% reduction in chance of death, and $18 and $21 for a 1% reduction in chance of side effects. The WTP to secure the benefit of antifungal prophylaxis was $2146 (95% CI $466, $4773) for parents and $735 (95% CI $75, $1397) for HCPs; there was a statistically significant difference in WTP between the parents and HCPs for antifungal prophylaxis (P<0.01). The marginal WTP for parents and HCPs were $316 and $269 for a 1% reduction in chance of infection, $1135 and $821 for a 1% reduction for chance of death, and $70 and $22 for a 1% reduction in chance of side effects.

## Discussion

We found that the chances of infection, death and side effects and costs all significantly influenced parents’ and HCPs’ utility, and that detail around administration may be more important to parents than providers. We also found that parents were willing to pay over twice the amount HCPs were willing to pay for prophylaxis. For antifungal prophylaxis, the statistically significant negative coefficient for the alternative specific constant was surprising given that most high risk patients receive some form of antifungal prophylaxis. We hypothesize that illustration of reasonable costs of antifungal prophylaxis, an intravenous option for medication administration, and lower risk of infection may have driven the negative preference for prophylaxis.

The difference in WTP was, in part, driven by the parameter estimates of the statistical model that revealed parents were willing to spend additional money on prophylaxis treatment compared to HCPs. The qualitative interview data in our companion manuscript revealed HCPs were heterogeneous in their attitudes regarding willingness to spend for prophylaxis treatment. For example, the interviews found that the differences between parent and HCP WTP may have been influenced by HCPs’ skepticism that parents could afford prophylaxis (particularly non-fluconazole antifungal prophylaxis) at higher drug prices. Further, HCPs highlighted that the additional money spent of prophylaxis may be detrimental to the health of the family because less money would be available for other goods such as food. Both statements suggest that HCPs would be conservative in their willingness to spend money on treatment prophylaxis (as borne by the quantitative results). Conversely, some interviewed HCPs suggested that cost of treatment should not be an issue because it may be covered through special access grants. This statement would have the effect of decreasing the parameter on the cost per 28 days of treatment attribute, which would increase WTP estimates. Further research into the heterogeneity of HCPs’ attitudes and preferences is warranted.

Studies that use DCE methodology to value WTP may be hindered because of the stated preference or hypothetical nature of the decision problem. This problem arises in DCEs because respondents are not bound by the choices they make. Furthermore, Canada has a universal healthcare system, and residents do not pay directly for most treatments. In health economics, the criterion validity of DCEs was initially demonstrated in a study that found those who had a positive WTP for a hip protector also elected to participate in a trial evaluating the hip protector. [12] Those who had a negative WTP chose not to participate in the trial. [12] Although these results are encouraging, more work on criterion validity is needed for DCEs in the Canadian context.

Our study has a number of limitations. The sample size in both of the DCEs was limited; this limitation makes robust, precise conclusions surrounding respondents’ WTP or comparisons between sub-groups for antibacterial or antifungal prophylaxis difficult. A second limitation is that we used parents and HCPs with experience in pediatric oncology. Some suggest that the preferences of members of the general public should be assessed in government-funded health care systems, since the public pays for health technologies through taxation. The value of antifungal and antibacterial prophylaxis in the context of societal preferences remains an area for future research. Two other limitations of our study are that we did not identify inconsistent responders and we did not measure dominance preference.

We did not include antimicrobial resistance as an attribute because our qualitative interview data suggested that participants did not think that this information was important for individual patient decision making. However, the exclusion of this attribute is an important limitation of our study. Some families and providers may indeed consider this issue when making decisions about prophylaxis. Furthermore, HCP preferences are likely to be influenced by institutional guidelines which will invariably consider the issue of antimicrobial resistance. This area merits further research.

The information from this study can help to determine prophylaxis strategies in the following ways. First, the data suggest that overall, parents will have a positive attitude to antibacterial prophylaxis and that once daily oral regimens will be best accepted. Second, programs that focus on patients at the highest risk of infection and death should be well received by parents and HCPs. Third, the WTP results provide some guidance as to what costs of prophylactic regimens will be considered acceptable. Finally, the derived WTP estimates can be used in cost-benefit analyses (where ‘benefit’ is measured in WTP) to examine issues surrounding value for money and the efficient allocation of scare health resources in the context of antibiotic prophylaxis in pediatric oncology. The cost benefit approach has been argued to be more robust compared to economic studies solely relying on naturalistic effectiveness outcomes [13]. This is because evaluations relying solely on naturalistic outcomes need to assume a range of WTP for an effectiveness gain to inform resource allocation [13]. Further, cost-benefit analyses can inform the efficient allocation of resources across all sectors of the economy, not just within a particular health system.

In summary, chances of infection, death, and side effects and costs were all significantly associated with utility for antibiotic prophylaxis. Parents have higher WTP for these strategies compared with HCPs. This knowledge can help to develop prophylaxis programs.

## Supporting Information

Appendix S1 **Visual representation of a choice experiment.**
(TIF)
