# Supplementary figures and images for: Discrete Choice Experiment to Evaluate Factors That Influence Preferences for Antibiotic Prophylaxis in Pediatric Oncology
Source: PLoS One. 2012 Oct 17;7(10):e47470. doi: 10.1371/journal.pone.0047470 (PMC3474806; doi:10.1371/journal.pone.0047470)

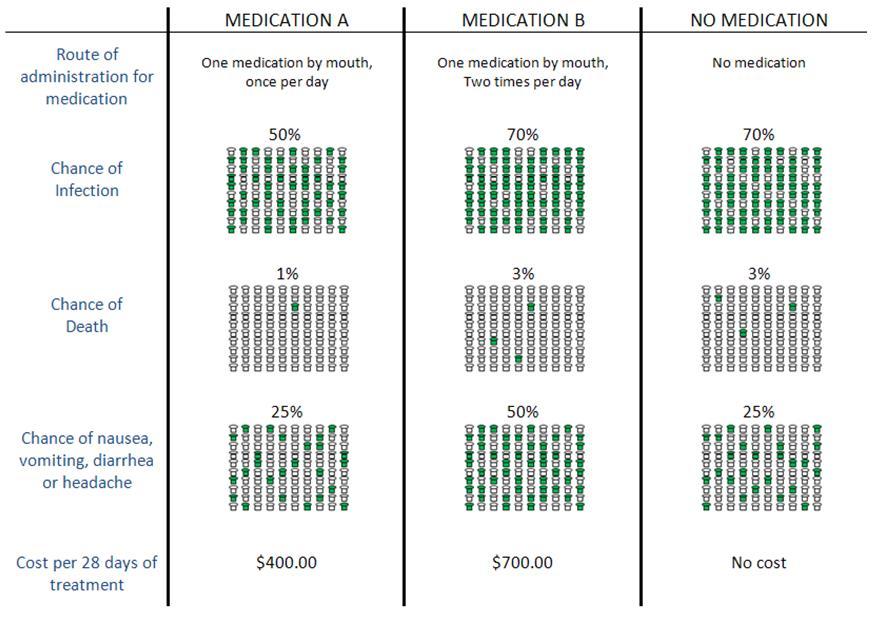

Supplement: Appendix S1 — Visual representation of a choice experiment. (TIF) [file pone.0047470.s001.tif]
